# “Salivary LINC00657 and miRNA-106a as diagnostic biomarkers for oral squamous cell carcinoma, an observational diagnostic study”

**DOI:** 10.1186/s12903-023-03726-0

**Published:** 2023-12-12

**Authors:** Nayroz Abdel Fattah Tarrad, Sandy Hassan, Olfat Gamil Shaker, Maha AbdelKawy

**Affiliations:** 1 Oral Medicine and Periodontology Department, Faculty of Dentistry, Fayoum University, Fayoum, Egypt; 2 Oral Medicine and Periodontology Department, Faculty of Dentistry, Fayoum University and Ahram Candian University, Fayoum, Cairo Egypt; 3 Medical Biochemistry and Molecular Biology Department, Faculty of Medicine, Cairo University, Cairo, Egypt; 4 Oral Medicine and Periodontology Department, Faculty of Dentistry, Beni-Suef University, Beni-Suef, Egypt

**Keywords:** Oral squamous cell carcinoma, Oral potentially malignant disorders, Saliva, LINC00657, miR-106a

## Abstract

**Background:**

Early detection and diagnosis of malignant tumors is critical for improving the survival rate and treatment outcomes of oral cancer. Thus, the current prospective investigation was designed to verify the role, sensitivity, and specificity of salivary LINC00657 and miRNA-106a as diagnostic markers in oral squamous cell carcinoma patients as compared to oral lichen planus (as an example of oral potentially malignant disorders) and normal individuals, and to show LINC00657 relation to miR-106a.

**Methods:**

A total of 36 participants were included, subdivided into 3 groups: Group I: 12 patients diagnosed with oral squamous cell carcinoma (OSCC). Group II: 12 patients diagnosed with oral lichen planus (OLP). Group III: 12 systemically free individuals with no oral mucosal lesions. Unstimulated salivary samples were collected from all participants to evaluate level of LINC00657 and miR-106a in different groups using quantitative real-time PCR.

**Results:**

OSCC showed the highest LINC00657 and lowest miR-106a fold change among included groups. Receiver Operating Characteristic (ROC) curve analysis of the two biomarkers for detecting OSCC revealed that LINC00657 had higher diagnostic accuracy (DA) (83.3%) compared to miR-106a (80.4%). As for detecting OLP, ROC analysis showed that miR-106a had higher (DA) (61%) compared to LINC00657 (52.5%). To discriminate OSCC from OLP, the diagnostic accuracy of both markers is the same (75%). Moreover, differentiating OSCC grades II and III, ROC analysis showed that miR-106a had lower (DA) (60%) compared to LINC00657 (DA) (83.3%).

**Conclusions:**

Salivary LINC00657 and miR-106a could be promising diagnostic markers for oral squamous cell carcinoma. Salivary LINC00657 may differentiate oral squamous cell carcinoma from oral potentially malignant disorders with considerable diagnostic accuracy. Moreover, low levels of salivary miR-106a could have the potential to indicate malignancy.

**Trial registration:**

The study was retrospectively registered on clinicaltrial.gov with NCT05821179 (first trial registration in 26/3/2023), date of registration: 19/4/2023.

**Supplementary Information:**

The online version contains supplementary material available at 10.1186/s12903-023-03726-0.

## Background

Worldwide, the 6th most common malignant tumors are oral and oropharyngeal tumors. Oral squamous cell carcinomas (OSCCs) comprise around 90% of head and neck tumors [1, 2].

OSCC may arise from pre-cancerous conditions known as oral potentially malignant disorders (OPMD) that are clinical lesions which have increased tendency to proceed into OSCC [3]. Leukoplakia, erythroplakia, oral lichen planus (OLP) as well as submucous fibrosis are considered among those OPMD [4].

Despite the high rate of mortality of OSCC, survival rates as well as prognosis could be better providing that oral cancer is detected and diagnosed early and that also may decrease its treatment morbidity [5]. Diagnosis of OSCC, when patient presents at clinic, is done in a late stage after becoming advanced and after occurrence of metastasis in approximately 50% of the cases [6]. This could be attributed to the fact that the majority of patients have no symptoms in early disease stages so do not ask for medical examination until the lesions become symptomatic [7]. Misdiagnosing malignant lesions as other inflammatory, benign, or reactive conditions may also result in delayed diagnosis and required treatment [8]. Consequently, finding a specific sensitive biomarker, that can differentiate between OSCC and OPMD and help early detection of OSCC by indicating which OPMD that is of great risk to turn malignant, will be of great clinical importance.

Single-stranded RNAs with a length of greater than 200 nucleotides are known as long non-coding RNA (lncRNA), the majority of them have not the ability of protein coding [9]. Through chromosomal modification, transcriptional activation / interference, LncRNA can have many physiological and biochemical cellular effects [10]. Research revealed that there is abnormal expression and regulation of lncRNA in many cancer types [11].

Long intergenic non-coding RNA 00657 (LINC00657) or otherwise called “NORAD” (non-coding RNA activated by DNA damage) has crucial effect on preserving genome stability and progression of cell cycle, therefore its dysregulation leads to cancer [12]. Up-regulation of LINC00657 has been reported in various tumors and was considered an indicator of poor survival, like breast cancer, gastric cancer, hepatocellular carcinoma, and lung cancer [13–16]. Moreover, elevated expression of LINC00657 indicated worse prognosis in OSCC patients and its knockdown decrease cell proliferation [17].

MicroRNAs (miRNAs), composed of nearly 21–25 nucleotides non-coding single-stranded RNAs, have a vital participation in regulation of expression of target genes through inhibiting translation of messenger RNA (mRNA) or stimulating its degradation [18]. Raising evidence have shown that miRNA dysregulated expression is linked to tumor initiation, development as well as cancer death via managing oncogene or tumor inhibitor gene [19–21]. miR-106a was found to act as a tumor suppressor in case of cancer bladder and renal cell carcinoma [22, 23]. A recent study had shown that miR-106a inhibited cell proliferation of OSCC cells in addition to inhibition of epithelial mesenchymal transition by directly lowering the expression of LIM kinase-1 [24].

Based on all that and relying on salivary markers as being non-invasive diagnostic modality we aimed in this study to investigate for the first time the significance of salivary LINC00657 and miR-106a as diagnostic biomarkers for OSCC as to the best of our knowledge the literature is lacking enough evidence on the role of salivary long non-coding RNAs in relation to oral cancer and OPMD.

## Methods

This prospective study as being an observational diagnostic study was approved by the research ethics committee of faculty of Dentistry Beni-Suef university (Approval number: # REC-FDBSU/06102022–03/AM), following the 1964 Declaration of Helsinki and its later amendments or comparable ethical standards. It included a total of 36 participants subdivided into 3 groups: Group I: 12 patients diagnosed with OSCC (6 females/6 Males) affecting different areas of the oral cavity with the majority located on tongue and being of grade II (*n* = 7) and III (*n* = 5) according to Broders’s criteria (grade I well- differentiated, grade II moderately- differentiated, and grade III poorly differentiated) [25]. Group II: 12 patients diagnosed with bullous erosive/atrophic oral lichen planus based on Andreasen’s classification [26] of OLP lesions into 6 clinical presentations (reticular, papular, erosive, atrophic, bullous, plaque) as an example of OPMD (6 females/6 males) showing dysplastic changes in 8 cases (4 mild, 4 severe dysplasia) [27] and no dysplasia in 4 cases. In addition, OLP lesions were mostly of bilateral distribution on buccal mucosa and tongue. Pain and ulcer scores were registered. Group III: 12 systemically free individuals with no oral mucosal lesions (7 females/5 Males). The patients represent a consecutive series and were recruited from the outpatient clinic of oral medicine and periodontology departments in Fayoum, Beni-Suef and Ahram-Candian universities on the time interval between October 2022 till February 2023.

All included individuals in this study signed written consent after clarifying to them the steps and aim of the study, in addition they were all subjected to medical and dental history taking along with clinical examination. Diagnosis of oral lesions from participants in group I and II was based on clinical examination and confirmed by biopsy (as the gold standard but with several disadvantages [28]) from lesional tissues via their histopathological evaluation [29] after coding of the samples with serial numbers. Surgical double wedge incisional biopsy [30] about 2 mm deep was done, from the most stained area of the lesion by toluidine blue stain, in an attempt to obtain the biopsy specimen.

### Eligibility criteria

1. The enrolled subjects were assessed medically according to modified Cornell Medical index [31] to ensure they were not suffering from any systemic disease, no pregnancy or lactation and not currently under any medication.
2. All subjects were clinically assessed and histopathologically confirmed to secure their categorization in the proper group where the control group had no oral mucosal lesions, the OLP group had atrophic/erosive lesions, and the malignant lesions included within the OSCC group.
3. Both gender and age within the range of 25–65 years old.

The current investigation aimed to verify the role of salivary LINC00657 and miRNA-106a as diagnostic markers in OSCC patients as compared to OLP and normal individuals, and to show LINC00657 relation to miR-106a consequently, unstimulated salivary samples were collected from all participants (diseased & control) utilizing standard techniques described by Navazesh [32] to evaluate the level of LINC00657 and miR-106a in different groups. Before salivary sample collection by ½ an hour the subjects were asked to withhold eating, smoking, or drinking. Collecting samples was performed in the morning by having the participants incline their heads forward to deliver saliva in a sterile test tube after swallowing. The obtained salivary samples were then preserved at − 20 °C until being evaluated. All samples were given codes and serial numbers before being sent for evaluation in the biochemistry lab.

ROC (Receiver Operating Characteristic) curve analysis was used to evaluate the diagnostic value of salivary LINC00657 and miR-106a for OSCC and OLP and their ability to discriminate between included groups.

### Real-time PCR for measurement of target miR-106 and LINC00657 expression in saliva

The levels of the miR-106 and LINC00657 were determined using quantitative real-time PCR (RT-qPCR). RT-qPCR was performed using the Rotor-gene Q real-time PCR system (Qiagen, USA). We used the RT2 SYBR Green PCR kit (Qiagen, MD, USA), a predesigned specific primer for both miRNA and lncRNA, and the housekeeping genes (SNORD-68 &GAPDH) were obtained from (Qiagen, Valencia, CA, USA).

The PCR thermal conditions for miRNA-106 were 15 min at 95 °C, 40 cycles of 15 s at 94 °C followed by 30 s at 55 °C and 30 s at 70 °C. The PCR cycling conditions for lncRNAs began with a 10-min incubation at 95 °C, followed by 40 cycles at 95 °C for 15 s and 60 °C for 60 s. The 2- ^ΔΔCt^ equation was used to calculate the salivary fold changes of miR-106 and LINC00657. Non-coding RNAs with a fold change (FC) less than one were downregulated, whereas those with an FC more than one were upregulated. The controls FC values were set as one [33].

### Sample size calculation

This power analysis used high and low expression of percentage of LINC00657 as the primary outcome. Based upon the results of XU et al. [17]; the proportions were 67 and 33% in patients with tumor stages T1-T2, 18 and 82% in patients with stages T3-T4. Using alpha (α) level of (5%), Power = 80%, the minimum estimated sample size was 8 subjects per group. Sample size calculation was performed using G*Power Version 3.1.9.2.

### Statistical analysis

Numerical data were explored for normality by checking the distribution of data and using tests of normality (Kolmogorov-Smirnov and Shapiro-Wilk tests). Age data showed normal (parametric) distribution while biomarkers data showed non-normal (non-parametric) distribution. Data were presented as mean, standard deviation (SD), median and range values. For parametric data, one-way ANOVA was used to compare the three groups. Bonferroni’s post-hoc test was used for pair-wise comparisons when ANOVA test is significant. For non-parametric data, Kruskal-Wallis test was used to compare between the three groups. Dunn’s test was used for pair-wise comparisons when Kruskal-Wallis test is significant. Qualitative data were presented as frequencies and percentages. Chi-square test was used to compare between the groups. ROC (Receiver Operating Characteristic) curve was constructed to determine the cut-off value for the two biomarkers to diagnose OSCC and OLP. ROC curve analysis was performed with MedCalc® Statistical Software version 19.5.1 (MedCalc Software Ltd., Ostend, Belgium; https://www.medcalc.org; 2020). Cut-off points were obtained from analyzed data and were not pre-specified. The significance level was set at *P* ≤ 0.05. Statistical analysis was performed with IBM SPSS Statistics for Windows, Version 23.0. Armonk, NY: IBM Corp.

## Results

### Participant flow chart: F. 1

**Fig. 1:**
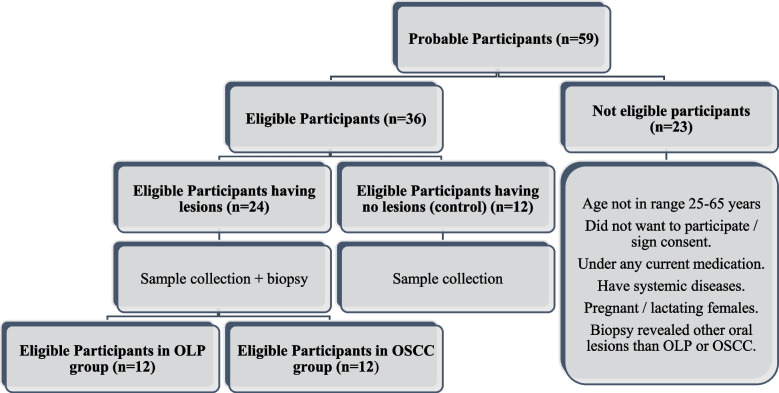
Initial examined number of participants and their categorization

The initial number of subjects evaluated in the present investigation was 59 individuals shown in Fig. 1 categorized to be included within the 3 study groups according to eligibility.

#### Base line characteristics

There was a statistically significant difference between mean age values in the three groups (Table 1). Pair-wise comparisons between the groups revealed that there was no statistically significant difference between OLP and control groups; both showed statistically significantly lower mean age values than OSCC group. There was no statistically significant difference between gender distributions in the three groups.

**Table 1:** Mean, standard deviation (SD), frequencies (n), percentages and results of one-way ANOVA test and Chi-square test for comparison between base line characteristics in the three groups

	OSCC (*n* = 12)	OLP (*n* = 12)	Control (*n* = 12)	*P*-value
Age (Years)				0.001*
Mean (SD)	53.1 (8) ^A^	41.2 (11.5) ^B^	38.7 (6.6) ^B^	
Gender [n (%)]				0.895
Male	6 (50%)	6 (50%)	5 (41.7%)	
Female	6 (50%)	6 (50%)	7 (58.3%)	

*: Significant at *P* ≤ 0.05, Different superscripts indicate statistically significant difference between groups

#### OSCC criteria

Criteria of OSCC are presented in Table 2. The most common site of OSCC was tongue (33.3%) and the least common sites were floor of the mouth and lip (8.3% for each site, respectively). The most common OSCC grade was Grade II in 58.3% of the cases. The most prevalent clinical stage was (T2 N0 M0 2) in 50% of the cases. Lymph node involvement was found in 16.7% of the cases while metastasis was found in only one case (8.3%).

**Table 2:** Frequencies (n) and percentages for OSCC criteria

OSCC Criteria	n	%
Site		
Tongue	4	33.3
Maxilla	2	16.7
Mandible	2	16.7
Hard palate	2	16.7
Floor of the mouth	1	8.3
Lip	1	8.3
Grade		
Grade II	7	58.3
Grade III	5	41.7
Clinical stage		
T1 N2 M0 4	1	8.3
T2 N0 M0 2	6	50
T3 N0 M0 3	4	33.3
T3 N1 M1 4	1	8.3
Lymph node involvement	2	16.7
Metastasis	1	8.3

#### OLP criteria

Criteria of OLP are presented in Table 3. All cases were medically free. The oral lesions were diagnosed as bullous erosive or atrophic types of OLP. The most common distribution of lesions was bilateral (66.7%) and the majority of cases show dysplastic changes (66.7%) with 50% showing mild dysplasia and 50% severe dysplasia. One case had affection of leg skin and one case had affected genitalia. As regards pain and ulcer scores, the median and range values were (8.0, 3-10) and (3.5, 2–5), respectively.

**Table 3:** Frequencies (n) and percentages for OLP criteria

LP Criteria	n	%
Medical/oral disease		
Free	12	100
Location		
Bilateral	8	66.7
Unilateral	4	33.3
Other affected sites		
Skin of the leg	1	8.3
Genitalia	1	8.3
Dysplasia		
Dysplastic changes	8	66.7
No dysplasia	4	33.3

#### Fold changes in miR-106a and LINC00657 biomarkers

The fold changes in both markers in different groups were shown in Table 4. As regards miR-106a: There was a statistically significant difference between fold changes of miR-106a in the three groups (*P*-value < 0.001, Effect size = 0.707). Pair-wise comparisons between groups revealed that the control group showed the statistically significantly highest fold change. The OLP group showed statistically significantly lower fold change. The OSCC group showed the statistically significantly lowest fold change.

**Table 4:** Descriptive statistics, results of Kruskal-Wallis test for comparison between fold changes of biomarkers in groups

Biomarker	Descriptive statistics	OSCC (*n* = 12)	OLP (*n* = 12)	Control (*n* = 12)	*P*-value	*Effect size (Eta Squared)*
miR-106a	Median (Range)	0.143 (0.042–0.439) ^C^	0.285 (0.121–1.444) ^B^	1.08 (0.87–1.39) ^A^	< 0.001*	0.707
	Mean (SD)	0.186 (0.124)	0.448 (0.384)	1.077 (0.162)		
LINC00657	Median (Range)	7.448 (2.243–28.432) ^A^	2.062 (1.106–27.284) ^B^	1.08 (0.9–1.31) ^C^	< 0.001*	0.222
	Mean (SD)	8.659 (7.179)	5.674 (7.727)	1.087 (0.121)		

*: Significant at *P* ≤ 0.05, Different superscripts in the same row indicate statistically significant difference between groups

##### While for LINC00657

There was a statistically significant difference between fold changes of LINC00657 in the three groups (*P*-value < 0.001, Effect size = 0.222). Pair-wise comparisons between groups revealed that OSCC group showed the statistically significantly highest fold change. The OLP group showed statistically significantly lower fold change. The control group showed the statistically significantly lowest fold change.

#### Diagnostic accuracy of the two biomarkers in detecting OSCC

**Fig. 2:**
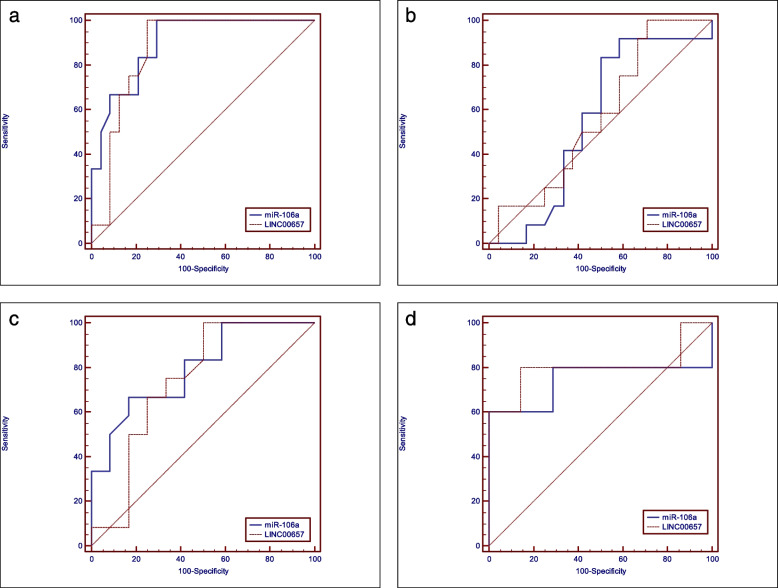
ROC curve of the two biomarkers for: (**a**) Detecting OSCC, (**b**) Detecting OLP, (**c**) Differentiation between OSCC and OLP and (**d**) Differentiation between OSCC Grades II and III

ROC curve analysis of the two biomarkers for detecting OSCC is presented in Table 5 and Fig. 2a. ROC curve analysis showed that LINC00657 showed higher diagnostic accuracy (83.3%) compared to miR-106a (80.4%). However, there was no statistically significant difference between the two biomarkers in detecting OSCC (*P*-value = 0.670).

**Table 5:** Cut-off values for LINC00657 & miR-106a and the corresponding sensitivity, specificity, predictive values, diagnostic accuracy, Area Under the ROC curve (AUC) and 95% confidence interval (95% CI) of the (AUC) with the two biomarkers to differentiate between different groups

Differentiation	Biomarker	Cut-off value	Sensitivity %	Specificity %	+PV %	-PV %	Diagnostic accuracy %	AUC	95% CI
OSCC from control	miR-106a	≤0.439	100	70.8	63.2	100	80.4	0.898	0.750–0.973
	LINC00657	> 1.376	100	75	66.7	100	83.3	0.870	0.716–0.958
OLP from control	miR-106a	≤0.557	83.3	50	45.5	85.7	61	0.554	0.379–0.719
	LINC00657	> 1.1	100	29.1	41.4	100	52.5	0.571	0.396–0.734
OSCC from OLP	miR-106a	≤0.168	66.7	83.3	80	71.4	75	0.795	0.582–0.931
	LINC00657	> 1.376	100	50	66.7	100	75	0.740	0.522–0.895
OSCC Grade II from Grade III	miR-106a	≤0.102	60	100	100	77.8	60	0.743	0.421–0.942
	LINC00657	> 9.353	80	85.7	80	85.7	83.3	0.8	0.48–0.967

+PV: Positive Predictive Value, −PV: Negative Predictive Value

#### Diagnostic accuracy of the two biomarkers in detecting OLP

ROC curve analysis of the two biomarkers for detecting OLP is presented in Table 5 and Fig. 2b. ROC curve analysis showed that miR-106a showed higher diagnostic accuracy (61%) compared to LINC00657 (52.5%). However, there was no statistically significant difference between the two biomarkers in detecting OLP (*P*-value = 0.824).

#### Diagnostic accuracy of the two biomarkers to differentiate between OSCC and OLP

ROC curve analysis of the two biomarkers for differentiation between OSCC and OLP is presented in Table 5 and Fig. 2c. ROC curve analysis showed that both biomarkers showed the same diagnostic accuracy (75%). There was no statistically significant difference between the two biomarkers (*P*-value = 0.629).

#### Diagnostic accuracy of the two biomarkers to differentiate between OSCC grades II and III

ROC curve analysis of the two biomarkers for differentiation between OSCC Grades II and III is presented in Table 5 and Fig. 2d. ROC curve analysis showed that miR-106a showed lower diagnostic accuracy (60%) compared to LINC00657 (83.3%). However, there was no statistically significant difference between the two biomarkers (*P*-value = 0.618).

#### Correlation between the two biomarkers (Table 6)

As regards OSCC as well as control groups, there was no statistically significant correlation between the two biomarkers. While in OLP group, there was a statistically significant inverse (negative) correlation between the two biomarkers (Correlation coefficient = − 0.615, *P*-value = 0.033).

**Table 6:** Results of Spearman’s correlation coefficient (*ρ*) for the correlation between the two biomarkers

Group	Correlation coefficient (*ρ*)	*P*-value
OSCC	−0.140	0.665
LP	−0.615	0.033*
Control	−0.063	0.846
Overall	−0.774	< 0.001*

*: Significant at *P* ≤ 0.05

As regards the overall correlation (regardless of group), there was a statistically significant inverse (negative) correlation between the two biomarkers (Correlation coefficient = − 0.774, *P*-value < 0.001).

## Discussion

As a result of the malignant process or its effect, cancer cells convey biochemical molecules known as tumor markers. Such tumor markers can be utilized as diagnostic or prognostic markers for patients suffering from cancer. In order to decrease cancer morbidity and mortality cancer detection as early as possible is of great significance [34, 35].

Saliva as a tool to screen large population serves as a cost-effective non-invasive method. Moreover, composition of saliva could throw light on many systemic disorders as well as physiological conditions with high precision drawing attention to salivary diagnostics value [35, 36]. lncRNA has critical role in cancer development and occurrence, in addition to cell metabolism and its development [37–39]. Consequently, this investigation was designed to clarify the role of salivary LINC00657 and miR-106a as non-invasive diagnostic biomarkers for OSCC and to verify if they can distinguish them from OPMD.

The current results revealed statistically significant difference between fold changes of both salivary LINC00657 and miR-106a in all groups. Although OSCC showed the highest fold change between the three groups regarding LINC00657, contrastingly it had the lowest fold change of miR-106a as compared to healthy and OLP groups. LINC00657 (NORAD) upregulation as a result of DNA damage in cancer is a common finding [40]. This role in cancer was achieved through inversely affecting PUMILIO proteins activity leading to instability of chromosome by inhibiting DNA repair and replication factors [41]. Silencing LINC00657 was found to obviously suppress OSCC cells proliferation capability while it could be promoted by the overexpression of LINC00657 [17].

Like our results concerning the significant decreased level of salivary miR-106a in OSCC samples compared to healthy group, a previous study found that significantly lower expression of miR-106a was present in OSCC tissues compared to the surrounding healthy tissues and confirmed that miR-106a upregulation dramatically suppress the proliferation of OSCC cells [24].

Moreover, in accordance with our results regarding increased expression of LINC00657 in OSCC samples compared to healthy group, Xu et al. [17] also found that LINC00657 was abnormally overexpressed in tissue samples of OSCC compared to adjacent healthy tissue samples and that it could allow OSCC malignant progression via the modulation of microRNA-150.

On this basis together with our findings we suggest a protective role for miR-106a in oral cancer unlike the probability of LINC00657 to have a carcinogenic effect in OSCC pathogenesis. Furthermore, the mentioned significant difference between OSCC and OLP groups in our study concerning the two markers may add another proof on the previous suggestion about their played part in the cancer scenario.

The data of the ROC analysis showed that the diagnostic accuracy of salivary miR-106a and LINC00657 for differentiating OSCC from healthy group was 80.4 and 83.3% respectively with 100% sensitivity for both markers, this could add evidence to their promising ability to be utilized as diagnostic markers for OSCC. This was also observed in studies of other cancers like colorectal cancer (CRC) where the overexpression of serum LINC00657 in cancer group was proved to have the capability of differentiating CRC from control and benign diseases and to be well thought out as a diagnostic marker for CRCs. Also, the same study revealed that serum miR106a decreased levels could predict the development of CRC in healthy participants [33] which likewise resembles the low expression of miRNA 106a in our cancer group salivary samples compared to healthy group and its high diagnostic accuracy in differentiating cancer and healthy groups.

Moreover, our results also revealed that salivary LINC00657 has high diagnostic accuracy (83.3%) regarding differentiating OSCC grade II and III unlike the low diagnostic accuracy concerning salivary miR-106a (60%) which highlight the ability of LINC00657 to indicate cancer aggressiveness and probability to spread. Similarly**,** Xu et al. [17], showed that LINC00657 overexpression reflected higher pathological stage indicating an overall lower survival rate.

Another investigation found that salivary lnc-PCDH9–13:1 was a specific and sensitive diagnostic marker for hepatocellular carcinoma [42] which may enforce that salivary lncRNAs could be a valuable non-invasive approach for cancer diagnosis.

The results herein showed that the diagnostic accuracy of both salivary markers for distinguishing OLP from healthy control were relatively low which could be explained by the presence of some OLP lesions without dysplasia.

In the current study the two markers revealed considerable diagnostic accuracy regarding their ability to differentiate malignant (OSCC) from potentially malignant lesions (OLP) with 75% accuracy for both markers. Although the correlation of the two markers was insignificant among the OSCC group which might be due to the small sample size of the study, there was a statistically significant inverse (negative) correlation between the two biomarkers regardless of the groups so reasonably this could propose that there is some sort of interaction between both markers. The role of LINC00657 and miR-106a is controversial in different types of cancers. A former investigation clarified that LINC00657 aberrant expression inhibited the growth of hepatocellular carcinoma cells through being a miR-106a-5p molecular sponge [43]. Accumulating data showed that LINC00657inhibiton could decrease human cancers development including esophageal squamous cell carcinoma [44] and pancreatic ductal adenocarcinoma [45]. We therefore hypothesize that overexpression of salivary LINC00657 along with downregulated levels of miR-106a might be linked in OSCC pathogenesis and might be required for a potentially malignant lesion to progress into OSCC via affecting the malignant progression of OPMD into OSCC.

In accordance with our results regarding the more older age group of OSCC patients than subjects in the other included groups, studies had previously indicated that older individuals are believed to have the highest risk to develop OSCC with more male predilection [46].

Our results showed low expression of salivary miR-106a in the OSCC group compared to OLP and control groups and that LINC00657 overexpression might have a way by which it could exert a down regulation of miR-106a expression. Moreover, salivary miR106a could be added to the list of tumor suppressor miRNAs for OSCC.

The small sample size of the present investigation, differences in mean age between groups together with not including other OPMD than OLP are among the study limitations. Studies with larger examined population and more age matched groups in addition to inclusion of other OPMD are recommended.

## Conclusions

Our study revealed that salivary LINC00657 and miR-106a could be sensitive non-invasive promising diagnostic markers for OSCC in relation to healthy subjects and that salivary LINC00657 may differentiate OSCC from OPMD with considerable diagnostic accuracy as well as its high diagnostic accuracy to distinguish different grades of OSCC. Besides, low levels of salivary miR-106a could have the potential to indicate malignancy. Future studies are needed to further analyze the mechanism which might be exerted by LINC00657 on regulation of miR-106a in tumorigeneses.

### Supplementary Information

**Additional file 1.**

## Data Availability

The datasets used and/or analysed during the current study are available from the corresponding author on reasonable request.

## Abbreviations

OSCCs: Oral squamous cell carcinomas
OPMD: Oral potentially malignant disorders
OLP: Oral lichen planus
lnc RNA: Long non-coding RNA
LINC00657: Long intergenic non-coding RNA 00657
NORAD: Non-coding RNA activated by DNA damage
miRNAs: MicroRNAs
mRNA: Messenger RNA
ROC: Receiver Operating Characteristic
RT-qPCR: Quantitative real-time PCR
CRC: Colorectal cancer
